# Nanomaterials and Coatings for Managing Antibiotic-Resistant Biofilms

**DOI:** 10.3390/antibiotics12020310

**Published:** 2023-02-02

**Authors:** Guillem Ferreres, Kristina Ivanova, Ivan Ivanov, Tzanko Tzanov

**Affiliations:** Grup de Biotecnologia Molecular i Industrial, Department of Chemical Engineering, Universitat Politècnica de Catalunya, Rambla Sant Nebridi 22, 08222 Terrassa, Spain

**Keywords:** nanoparticles, quorum sensing, quorum quenching, antiadhesion, antimicrobial, biofilm inhibition

## Abstract

Biofilms are a global health concern responsible for 65 to 80% of the total number of acute and persistent nosocomial infections, which lead to prolonged hospitalization and a huge economic burden to the healthcare systems. Biofilms are organized assemblages of surface-bound cells, which are enclosed in a self-produced extracellular polymer matrix (EPM) of polysaccharides, nucleic acids, lipids, and proteins. The EPM holds the pathogens together and provides a functional environment, enabling adhesion to living and non-living surfaces, mechanical stability, next to enhanced tolerance to host immune responses and conventional antibiotics compared to free-floating cells. Furthermore, the close proximity of cells in biofilms facilitates the horizontal transfer of genes, which is responsible for the development of antibiotic resistance. Given the growing number and impact of resistant bacteria, there is an urgent need to design novel strategies in order to outsmart bacterial evolutionary mechanisms. Antibiotic-free approaches that attenuate virulence through interruption of quorum sensing, prevent adhesion via EPM degradation, or kill pathogens by novel mechanisms that are less likely to cause resistance have gained considerable attention in the war against biofilm infections. Thereby, nanoformulation offers significant advantages due to the enhanced antibacterial efficacy and better penetration into the biofilm compared to bulk therapeutics of the same composition. This review highlights the latest developments in the field of nanoformulated quorum-quenching actives, antiadhesives, and bactericides, and their use as colloid suspensions and coatings on medical devices to reduce the incidence of biofilm-related infections.

## 1. Introduction

Biofilms are structured and coordinated communities of microbial cells on a surface. They are formed by initial attachment of bacterial cells, followed by proliferation and enclosure in a self-produced extracellular polymeric matrix (EPM), which is composed mainly of water (97%), exopolysaccharides, proteins, and nucleic acids [1]. The EPM formation is triggered by an intercellular communication process, known as quorum sensing (QS) [2]. During this process, bacteria secrete signaling molecules, acyl homoserine lactones (AHLs), or autoinducer peptides (AIPs) in the case of Gram-negative and Gram-positive bacteria, respectively, generally termed autoinducers (AIs) [3]. When the bacterial population reaches a certain density, which in turn leads to a certain threshold level of AIs, genes for biofilm formation and production of virulence factors are expressed [4].

Bacterial biofilms are closely related with antimicrobial resistance (AMR), which is a global concern for increased morbidity and mortality, prolonged hospitalization, and additional financial costs. A high number of bacterial species are already resistant to most of the existing antibiotics due to acquired molecular mechanisms allowing the expulsion, inactivation, or destruction of antimicrobials [5,6]. Thereby, the biofilm mode of growth is even more challenging. The formation of a densely packed EPM makes the cells 100 to 1000 times less susceptible to antibacterials than their free-floating counterparts and bacteria encased in biofilms exhibit higher tolerance to the host immune response [7,8,9]. The EPM holds the pathogens together, protects them, and reduces the diffusion of drug molecules, while the intimate biofilm environment offers excellent conditions for horizontal gene transfer and further resistance development [10,11]. Consequently, several mechanisms, such as limited drug penetration, decelerated bacterial growth, and expression of specific protective factors are recognized for the pronounced resistance of biofilms [12]. The formation of biofilms is responsible for numerous acute and chronic diseases such as cystic fibrosis, otitis, endocarditis, chronic wounds, periodontitis, and dental caries and their treatment frequently involves intensive antibiotic treatment and a combination of various antibiotics at high dosages, further aggravating the AMR issue [13,14,15]. The Gram-negative *Escherichia coli*, *Klebsiella pneumoniae*, *Pseudomonas aeruginosa*, and *Proteus mirabilis*, and the Gram-positive *Enterococcus faecalis*, *Staphylococcus aureus*, *Staphylococcus epidermidis*, as well as the yeast *Candida albicans*, are among the most common biofilm-forming species, found in up to 60% of severe and persistent hospital-acquired infections [16]. In addition, the frequent usage of indwelling devices leads to even more nosocomial infections [17]. Furthermore, biofilms have a detrimental impact on a wide range of industrial sectors such as food packaging, water filtration systems, marine equipment, and industrial bioreactors [18,19,20,21,22].

Given the growing impact of AMR, there is an urgent need to develop novel antibacterial strategies in order to outsmart the bacterial evolutionary mechanisms. Effective strategies for prevention and treatment of bacterial infections with a lower risk of AMR should integrate: (i) bacterial eradication without creating selective pressure [23], (ii) prevention and elimination of biofilm formation [24,25], (iii) biocompatibility, protection of the beneficial strains and host environment [26], (iv) long-term stability [27].

Recently, nanotechnology-based approaches have gained immense attention because the nano form endows the actives with stability and enhanced antimicrobial efficacy compared to the same material in bulk form [28,29]. Nanomaterials and nanoformulation have been employed to give a new life to obsolete antibiotics, specifically, to deliver different actives to the site of infection or act as a structural and/or functional element in medically relevant materials and coatings. Nanoparticles (NPs) have been deposited on medically relevant surfaces, medical devices, and implants by techniques like layer-by-layer, sonochemistry, and spin coating to engineer durable nanostructured coatings against biofilm formation [30,31,32]. Apart from eradicating planktonic cells and preventing biofilm formation, nano-sized actives are also highly potent in the elimination of already established drug-resistant biofilms. Thus, NPs and small vesicles have been used as delivery vehicles of biofilm dispersants (e.g., matrix-degrading enzymes, NO-donors) or direct bactericidals (e.g., antibiotics, antimicrobial peptides) [33,34,35,36]. In this review, we attempt to highlight the latest advances in the engineering of functional nano-enabled materials and coatings to combat bacterial biofilms and the spread of resistance. The use of nano-formulated enzymes, natural compounds, and their synthetic mimics able to scramble the wires of bacterial communication before it starts and consequently, to attenuate bacterial virulence and biofilm formation is discussed. The design of innovative nano-actives targeting inhibition of the initial bacterial settlement and destabilization of the protective EPM is also outlined. Finally, recent attempts in the nanoformulation of other non-antibiotic actives with broad-spectrum antibacterial activity, increased safety, and low-resistance-inducing mechanisms of action are described as prominent alternatives for managing biofilm-related infections (Table 1).

## 2. Anti-Virulence Approaches for Managing Biofilm Infections

**Figure 1:**
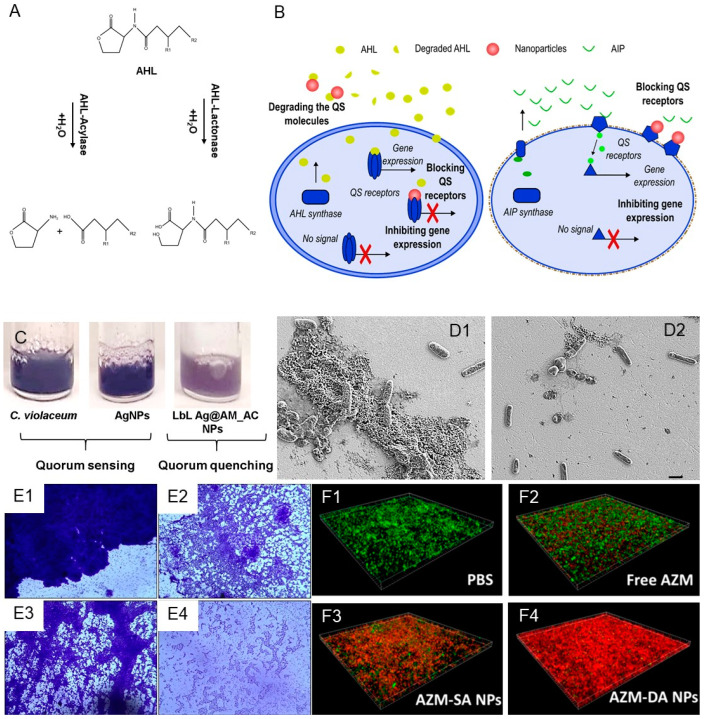
(**A**) Degradation of AHLs signals by quorum-quenching enzymes lactonase and acylase. (**B**) Schematic representation of QS process in Gram-positive and Gram-negative bacteria and different mechanisms for its inhibition. Gram-negative bacteria produce acyl-homoserine lactones (AHLs, yellow circles), while Gram-positive secrete autoinducer peptides (AIPs, green lines). When the concentration of these molecules reaches a certain threshold, the genes related with virulence and biofilm formation are expressed. Nanoformulated QQE, QSI, or metals are employed to degrade the QS signals outside the cells or block the cognate QS receptors in bacteria. (**C**) Inhibition of bacterial virulence by Ag NPs coated with aminocellulose and acylase I assessed through the decrease in the QS-regulated violacein production by *C. violaceum* (reproduced from [33] under the terms of the Creative Commons Attribution International License (CC BY 4.0)). (**D**) SEM images of untreated *P. aeruginosa* biofilm (**D1**) and treated with acylase loaded NPs (**D2**) (reproduced from [28] under the terms of the Creative Commons Attribution International License (CC BY 4.0)). (**E**) Light microscopic images of the untreated *P. aeruginosa* (**E1**) and *S. marcescens* MTCC 97 biofilms (**E3**), and in the presence of AuNPs synthesized using *C. annuum* extract (**E2** and **E4**, respectively) (reproduced from [59] under the terms of the Creative Commons Attribution International License (CC BY 4.0)). (**F**) Live/dead staining assay of *P. aeruginosa* biofilms (**F1**) treated with free azithromycin (**F2**), and in its nanoform (**F3** and **F4**) (Reprinted with permission from Gao et al., ‘Size and Charge Adaptive Clustered Nanoparticles Targeting the Biofilm Microenvironment for Chronic Lung Infection Management.’ *ACS Nano* 2020, 14, 6588–5699. Copyright 2020 American Chemical Society [45]).

Targeted interruption of the cell-to-cell communication with quorum-quenching enzymes (QQE), which degrade bacterial messengers, or by chemical QS inhibitors (QSIs), inactivating specific receptors, is an innovative and effective way to tackle the challenges of bacterial infections. Disturbing the QS lowers the expression of genes for synthesis of virulence factors and adhesive components of the EPM without affecting the bacterial viability, thus exerting low selective pressure for mutation (Figure 1). However, the practical use of the anti-QS compounds is frequently limited due to their potential toxicity, low stability and reduced therapeutic efficacy compared to antibiotics. In order to address these limitations, significant efforts have been devoted to nanoformulation.

### 2.1. Nano-Formulated Quorum-Sensing Inhibitors

Some of the most widely used molecules that inhibit or obstruct the QS machinery are cinnamaldehyde, quercetin, azithromycin, or eugenol (Table 1). Cinnamaldehyde, an aromatic compound present in cinnamon, is able to disrupt the autoinducer-2 system of *S. aureus* and the LuxR-mediated transcription from the *P_luxI_* promoter in Gram-negative bacteria [37]. This compound has been incorporated in gold NPs using a silica coating and encapsulated inside chitosan NPs, which improved its effectivity compared to the bulk form [38,39]. The plant flavonoid quercetin has also been encapsulated in chitosan NPs and combined with silver NPs [40,41,42]. Azithromycin is an antibiotic that has been reported to inhibit the synthesis of AHLs at sub-inhibitory concentrations and it has been introduced in hyaluronic acid-poly(lactic-*co*-glycolic acid) nanovesicles in order to deliver the drug inside the biofilm [43,44]. Similar results were obtained by Gao et al. after combining this active with cationic polymers—the resulting NPs were able to penetrate the biofilm and eradicate mature *P. aeruginosa* biofilm in vitro and in vivo in a lung infection model (Figure 1F) [45]. Finally, eugenol is a catechol, obtained from clove, cinnamon leaf, pimento, bay, sassafras, massot bark oils, that has been reported to reduce the biofilm metabolic activity due to QS disruption in Gram-negative strains, inducing biofilm detachment [46]. Different types of eugenol nanoemulsions have been successfully generated and showed a capacity to inhibit the production of AHLs and biofilm formation in *P. aeruginosa*, protect surfaces when applied as hydrogel coatings, and improve the treatment outcomes of wound infection when loaded in a dressing with silver [47,48,49,50,51].

### 2.2. Quorum-Quenching Enzyme NPs

Two hydrolytic enzymes, which degrade AHLs—acylase and lactonase—have been widely exploited to inhibit QS-regulated virulence and biofilm growth in Gram-negative bacteria [52] (Figure 1A and Table 1). In our group, we employed acylase in combination with silver NPs, which were coated with aminocellulose and enzyme in a layer-by-layer fashion, adding a membrane-disturbing functionality. The obtained hybrid NPs reduced the QS indicator violacein in *Cromobacterium violaceum* and inhibited the planktonic growth and biofilm formation of *P. aeruginosa* at lower NP concentrations, non-toxic to human cells (Figure 1C) [33]. In another work, we nanoformulated acylase with the conventional antibiotic gentamicin in order to boost the bactericidal activity of the latter, while conferring biofilm inhibition activity on *P. aeruginosa* (Figure 1D) [28]. Other researchers have combined acylase with graphene oxide and mesoporous silica NPs for antifouling coatings of membranes [53,54]. In parallel, lactonase that catalyzes the breaking of ester bonds in the lactone ring has been nanohybridized with gold and silver to inhibit the biofilm production of *Proteus* species and *K. pneumonia*, respectively [55,56,57]. The extracellular disturbing of QS is among the most promising anti-QS approaches because it avoids the need to penetrate the cells or reach their receptors. However, despite the great potential of enzymes, their activity in vivo might be compromised; hence, further studies are needed to validate their antibiofilm activity in relevant environments.

### 2.3. Metal NPs as QS Inhibitors

Metal (e.g., silver, copper) and metal oxide (e.g., zinc oxide and copper oxide) NPs are gaining attention due to their strong antibacterial efficacy and lower probability for inducing AMR. Recent evidence has demonstrated that apart from direct killing, metal NPs may also interfere with bacterial community behavior and act as QS inhibitors (Table 1). For instance, silver NPs with an average size of 20–40 nm at concentrations of 10–25 µg/mL reduced the biofilm formation and inhibited the virulence of *P. aeruginosa* [58]. Gold NPs, produced using *Capsicum annuum* as reducing and active agent, impeded the biofilm development of *P. aeruginosa* and *Serratia marcescens*. The authors speculated that inhibition of the synthesis of QS signals and blocking of regulatory proteins is a possible QQ mechanism (Figure 1E) [59]. Nickel oxide NPs have presented hydrolase-like activity by degrading AHLs, which inhibited the violacein production in *C. violaceum* and biofilm formation in *P. aeruginosa* [60]. Copper NPs, coated with polyacrylic acid, were able to downregulate the expression of *ppyR*, which is related with the regulation of *Psl* operon that promotes bacterial adhesion of *P. aeruginosa* [61]. In another work, the anti-QS potential of selenium and tellurium NPs was also demonstrated. An up to 80% decrease in the QS-regulated violacein expression and a significant reduction of *P. aeruginosa* biofilm were obtained for both NPs types [62]. Zinc oxide NPs and, to a lesser degree, titanium oxide NPs, also displayed an ability to interfere with the AHL system of *C. violaceum* [63].

### 2.4. Mimicks of QS Signals and Inhibitors

Apart of the aforementioned direct anti-QS strategies, it is worth mentioning the design of more advanced approaches, exploiting the sequestration, camouflaging, and mimicking of QS machinery (Table 1). For example, Lu et al. downregulated the production of virulence factors and impeded biofilm production of *Vibrio cholera* with NPs, delivering the bacterium autoinducer CAI-1 at high concentrations [64]. In another approach, computationally designed polymers were used to sequester QS molecules of Gram-negative bacteria and consequently alter the QS-controlled phenotype [65]. However, the efficacy of the latter approach is restricted by the adsorption capacity of the polymer that, upon saturation, may instead act as a source for potentiating the QS. To address this issue, Garcia Lopez et al. generated innovative molecularly imprinted NPs able to mimic the catalytic activity of lactonases [66]. In another work, micellar imprinting of AHL-like templates yielded NPs, in which acidic zinc hydrolyzed the acyl chains of C8-AHLs [67].

## 3. Antifouling, Antiadhesive, and Biofilm-Dispersing Nanoactives

**Figure 2:**
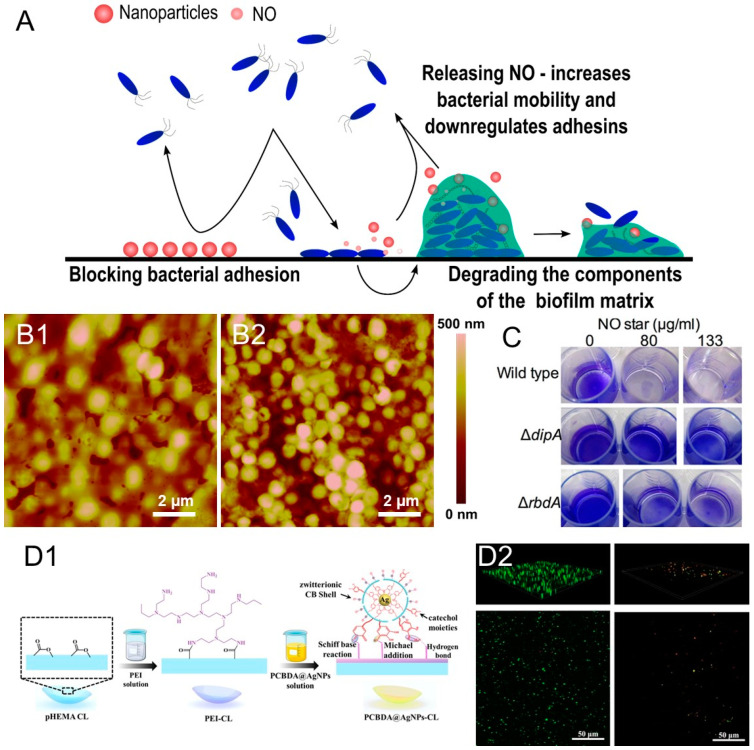
(**A**) Schematic representation of the anti-adhesive and biofilm-dispersing strategies covered in this review. (**B**) Nanoaggregates of Ag-amylase degraded EPM of *S. aureus* biofilm on gold disks. AFM images of the disk showing EPM matrix (**B1**) and its disintegration by the hybrid Ag-amylase NPs (**B2**) (reproduced from [34] under the terms of the Creative Commons Attribution International License (CC BY 4.0)). (**C**) Cristal violet staining of *P. aeruginosa* biofilms treated with different concentrations of NO-core cross-linked star (Adapted with permission from Duong et al., ‘Nanoparticle (Star Polymer) Delivery of Nitric Oxide Effectively Negates *Pseudomonas aeruginosa* Biofilm Formation’. *Biomacromolecules* 2014, 15, 2583–2589. Copyright 2014 American Chemical Society [71]). (**D**) Scheme of PCBDA@Ag NPs deposition on contact lenses (**D1**), and confocal microscopy of *P. aeruginosa* grown on pristine and coated contact lenses (**D2**) (Reprinted from J. Colloid Interface Sci, 610, Ma et al., Commercial Soft Contact Lenses Engineered with Zwitterionic Silver Nanoparticles for Effectively Treating Microbial Keratitis 923–933, Copyright 2022, with permission from Elsevier [79]).

The anti-adhesion strategy comprises prevention of the initial bacterial adherence to the surface or detachment of already established biofilms. Inhibiting the initial bacterial adhesion by preventing the contact of surface with the bacterial cells or their anchoring components, impeding the transition from motile to sessile cells via alteration of the bacterial mechanism, or targeting the adhesive and anchoring components of the EPS are also among the most promising strategies to maintain bacteria in planktonic form (Figure 2 and Table 1).

### 3.1. Nitric Oxide Donors Loaded in Nanocarriers

Nitric oxide (NO) is an endogenous molecule that has gained increasing attention due to its potential to impede bacterial adhesion and biofilm occurrence. NO is able to reduce the levels of the intracellular cyclic-di-GMP messenger, promoting the dispersion of biofilms [68]. Although its mechanism of action is still under investigation, there is evidence that NO enhances the activity of phosphodiesterases, upregulates genes for motility, and downregulates the expression of virulence factors and adhesins. However, due to its high reactivity, its direct application as gas is not feasible. Several NO delivery nanosystems have been developed in order to drive NO in a safe and effective way to the specific site of infection because NO has a short half-life and rapidly diffuses from the release site [69]. NO-releasing silica NPs demonstrated the capacity to kill *P. aeruginosa*, *E. coli*, *S. aureus*, and *S. epidermidis* encased in biofilms [70]. Duong et al. described how polymeric-star NPs, conjugated with spermine and NO, hindered the transition from swimming bacteria to sessile and dispersed *P. aeruginosa* biofilm, subsequently increasing the number of released planktonic cells in suspension (Figure 2C) (Table 1) [71]. In addition, Slomberg et al. demonstrated that the size and shape of the NO carrier play an important role in its release, small rod-shaped delivery vehicles being the most effective [72]. NO donors have been loaded into poly(vinyl alcohol) and poly(ethylene glycol) films and alginate hydrogels together with silver NPs for antibacterial topical applications (Table 1) [73,74]. Silica NPs loaded with nitroprusside acted synergistically with ampicillin and tetracycline, improving their bactericidal effect towards *S. aureus* and *S. epidermidis* [36].

### 3.2. Zwitterionic Materials

Zwitterions hold an immense potential for antifouling due to the formation of a hydration layer and steric hindrance effect that impedes the contact and adherence of the fouling biomolecules (e.g., proteins, polysaccharides) [75]. An essential step in the biofilm formation is the ability of bacteria to colonize surfaces via unspecific interaction of their surface proteins that serve as primary anchoring points. The equimolar number of homogenously distributed positively and negatively charged moieties along the zwitterion chain allows the binding of water molecules, creating a barrier towards protein attachment and bacterial colonization [76]. Poly(sulfobetaine methacrylate) (PSBMA) has been combined with silver NPs, which reduced the adhesion of proteins and inhibited *P. aeruginosa* biofilm formation on thin-film composite membranes under dynamic flow (Table 1) [77]. Xin et al. conjugated silver NPs with modified sulfobetaine in polyester membranes against *E. coli* and *S. aureus*, while Ma et al. combined NPs with poly(carboxybetaine-co-dopamine methacrylamide) (PCBDA) to coat contact lenses that reduced corneal infection in a rabbit model (Figure 2D) [78,79]. Xiang et al. immobilized PCBDA-Ag NPs on cotton gauzes, which not only inhibited bacterial adhesion and biofilm formation, but also promoted wound healing (Table 1) [80].

Antifouling surfaces and materials aim to prevent the first stage of the biofilm growth, but they do not kill the bacteria and the latter are able to colonize other surfaces and tissues. Hence, this anti-biofilm strategy may be feasible for material applications of short duration such as sutures, meshes, and drainage tubes, but not in, e.g., long-term implants.

### 3.3. Nanoformulated Matrix-Degrading Enzymes

Matrix-degrading enzymes target the extracellular components secreted by bacteria during biofilm formation. The polysaccharide, protein, and nucleic acid components of the EPS provide diverse benefits for bacteria. Carbohydrates play an important role in the anchoring of sessile cells, while proteins provide structural stability of the three-dimensional biofilm structure. Some of the proteins are catalytically active and possess the ability to digest large biomolecules (e.g., glycoside hydrolases and lipases) to supply nutrients, or take part in redox reactions (e.g., catalase). Nucleic acids also participate in the adhesion, aggregation, and cohesion of the biofilm, but their characteristic role is focused on the transfer of genetic information [81].

Degrading the biofilm adhesive structure by different enzymes such as α-amylase, alginate lyase, proteases, and deoxyribonucleases (DNases) is a feasible strategy to weaken biofilms and increase the bacterial susceptibility to antibacterial agents. α-amylase is a glycoside hydrolase that catalyzes the breaking of α-1,4-glycosidic bonds. Its main substrate is starch; however, it also acts on several carbohydrates present in the biofilm matrix [82]. We have developed NPs containing α-amylase and silver via gallic acid/laccase-mediated crosslinking, which were able to eradicate already established biofilms of *P. aeruginosa* and *S. aureus* (Figure 2B and Table 1) [34]. In another study, we hybridized α-amylase and zinc oxide in an NP form to coat urinary catheters in a single-step sonochemical approach. Thus, the undesirable phenomenon of unspecific protein adsorption was turned into an advantage by using the enzyme as adhesive. The resulting nanostructured coating hindered the formation of *S. aureus* and *E. coli* biofilms in a model of a catheterized bladder and significantly reduced the incidence of bacteriuria in rabbit models [31]. Abeleda et al. employed amylase to design hybrid silver/amylase NPs that inhibited and eradicated *K. pneumonia* and resistant *S. aureus* biofilms [83]. Alginate lyase is another biofilm-dispersing enzyme that breaks down polysaccharides, which are responsible for the strong adhesion and resistance of *P. aeruginosa* biofilms [84]. The enzyme was immobilized on chitosan and silver NPs, enhancing the effectivity of the conventional antibiotics ciprofloxacin and ceftazidime [85,86,87]. Proteases also have been nanoformulated using different approaches. For example, antibiotic-loaded shellac NPs, coated with the biofilm-degrading serine endo-peptidase alcalase, reduced the protein concentration in *S. aureus* EPM and effectively eradicated bacterial cells in the biofilm (Table 1) [88]. Antibiotic-carrying nanogels functionalized with antibiofilm protease were effective towards *Staphylococcus aureus*, *Pseudomonas aeruginosa*, *S. epidermidis*, *K. pneumoniae*, *E. coli*, and *E. faecalis*, while conjugating the enzyme with gold nano-rods completely eradicated *S. aureus* and *E. coli* biofilms under photothermal treatment [89,90].

Deoxyribonuclease I (DNAse I), which can degrade DNA in the biofilm matrix, has also shown a negative impact on the biofilm structure of medically relevant pathogens such as *P. aeruginosa*, *E. coli*, *S. aureus*, and *K. pneumonia*. DNAse I was integrated with ciprofloxacin to disperse already established *P. aeruginosa* biofilms and increase the antibiotic susceptibility. Its combination with silver NPs boosted the bactericidal effect and acted effectively against *P. aeruginosa*, *Streptococcus mutans*, and *E. coli* biofilms [91,92]. Combinations of several matrix-degrading enzymes have been shown to achieve better results in compromising the ability to establish or maintain stable biofilm structure. Protease and DNase have been loaded in liposomes to disperse *Cutibacterium acnes* biofilms in vitro and maintained their inhibition capacities in skin and catheter models without affecting cell viability in mice [93]. Although these enzyme-based approaches show very promising results, the major limitation is related to catalyst inactivation. Furthermore, the biofilm dispersion capacity is influenced by the nature of the species, which produce different matrix components, so a one-size-fits-all solution may not be feasible [94].

## 4. Antimicrobial Nanoactives

**Figure 3:**
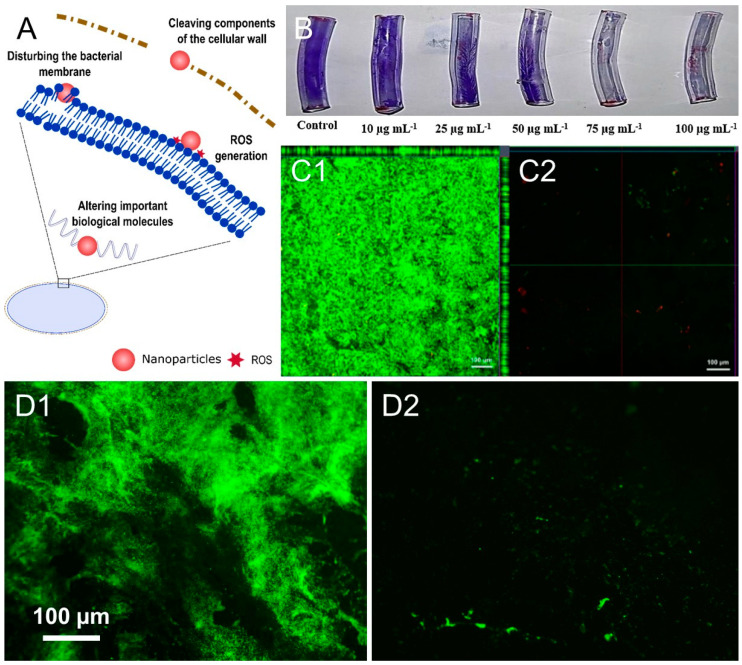
(**A**) Schematic representation of the antimicrobial mechanisms of action of bactericidal NPs covered in this review. (**B**) Cristal violet staining of *P. aeruginosa* biofilm grown on catheters coated with different concentrations of AgNPs (Reprinted from Prog. Org. Coati, 151, LewisOscar et al., ‘In vitro analysis of green fabricated silver nanoparticles (AgNPs) against *Pseudomonas aeruginosa* PA14 biofilm formation, their application on urinary catheter,’ 106058, Copyright 2021, with permission from Elsevier [126]). (**C**) Confocal microscopy images of untreated biofilm (**C1**) and treated with chitosan-cellobiose dehydrogenase-DNase NPs (**C2**) (Reprinted from Mater. Sci. Eng. C, 108, Tan et al., ‘Co-Immobilization of Cellobiose Dehydrogenase and Deoxyribonuclease I on Chitosan Nanoparticles against Fungal/Bacterial Polymicrobial Biofilms Targeting Both Biofilm Matrix and Microorganisms,’ 110499, Copyright 2019, with permission from Elsevier [124]). (**D**) Fluorescence microscopy images showing the grown of *P. aeruginosa* biofilm on pristine silicone catheters (**D1**) and its inhibition when the catheters were coated with self-assembled pSBMA-polymyixin B NPs (**D2**) (reproduced from [100] under the terms of the Creative Commons Attribution International License (CC BY 4.0)).

Biofilms reduce the susceptibility of bacteria to conventional antibiotics due to restricted diffusion in the EPS; however, nanoformulation may enhance the penetration. In addition, other compounds with wide spectrum and low-resistance-inducing mechanisms of action overcome the main limitations of conventional drugs. In this section, we focus on actives that disrupt the bacterial membrane, such as peptides and lipids, enzymes, which damage the cell wall and produce reactive oxygen species (ROS), and metal NPs with several antibacterial mechanisms, including ROS generation and release of toxic ions (Figure 3).

### 4.1. Antimicrobial Peptides

Antimicrobial peptides (AMP) are small oligopeptides of five to a hundred amino acids, found in bacteria, protozoa, fungi, plants, insects, and animals. AMPs form pores in the microbial membranes, which enables eradication of a broad spectrum of microorganisms and is less likely to lead to AMR. Despite the pronounced antimicrobial activity, even against multidrug-resistant strains, the low stability of AMP in biological fluids limits their practical application. To overcome this issue, different nanoformulation and hybridization strategies have been proposed. RBRBR was loaded into chitosan NPs and inhibited the formation of biofilm by reducing the number of viable motile bacteria (Table 1) [95]. Seferji et al. photoionized IVFK in order to reduce silver and form nanocomposites, which acted against *S. aureus* and *E. coli* biofilms [96]. Zhang et al. produced NPs by self-assembly of pHly-1 at acidic conditions. These peptide NPs were able to kill *S. mutans* in acidic cariogenic biofilm microenvironment displaying interesting properties for odontology applications [97]. The simultaneous nanoformulation of AMP and antibiotics leads to synergistic activity for eradication of *P. aeruginosa*. Yu et al. engineered stimuli-responsive NPs loaded with melittin and ofloxacin [98]. Gupta et al. combined AMP with quaternary ammonium polymers and the resulting NPs were able to penetrate biofilm and kill sessile *S. aureus*, *P. aeruginosa*, and *Enterococcus cloacae* [99]. In our group, we synthesized and deposited on silicone catheters peptide-zwitterion NPs via a one-step sonochemical approach. Thereby, catheters were incubated with polymyxin B and PSBMA under ultrasound and the formed NPs were coated through a “throwing stones” process. The coated silicones presented contact killing and antibiofilm properties without affecting mammal cells and were able to eliminate 80% of *P. aeruginosa* biofilms (Figure 3D and Table 1) [100]. Although the potent wide-spectrum antimicrobial activity of AMPs predicates them as a promising tool, their accumulation can lead to unspecific toxicity and they also suffer from stability issues in biological fluids once released from the nanocarrier [101].

### 4.2. Non-Peptide Mimics of AMPs

Non-peptide cationic steroids with enhanced stability, termed ceragenins, have been developed to overcome some of these drawbacks. Their molecular structure is based on cholic acid, appended by amine groups, which are arranged to reproduce the amphiphilic morphology of AMPs. These AMP mimics act on a variety of Gram-positive and Gram-negative pathogens, including multi-drug resistant strains in planktonic and sessile forms, but unlike AMPs, they demonstrate enhanced stability at physiological conditions and lower toxicity [102,103,104]. Due to the high positive charge and amphipathic nature, ceragenins interact with negatively charged cell membranes causing changes in the organization of the membrane lipid bilayer, change of permeability, and cell death. They have also the ability to bind bacterial endotoxins (e.g., lipopolysaccharides and lipoteichoic acid), which is further translated into anti-inflammatory effects [105]. Recently, Paprocka et al. demonstrated the benefit of using synergistic combinations of ceragenins with commercial antibiotics including ceftazidime, levofloxacin, co-trimoxazole, and colistin for managing of *Stenotrophomonas maltophilia* infections. Ceragenins applied simultaneously with β-lactam antibiotics (e.g., ceftolozane/tazobactam, ceftazidime/avibactam, meropenem/vaborbactam) at low concentrations also showed efficacy against clinical strains of *P. aeruginosa* regardless of their resistance mechanisms [106]. Rod, peanut, and star-shaped gold NPs conjugated with ceragenins exerted potent bactericidal activity against multi-drug resistant strains due to the generation of ROS, followed by membrane damage and the leakage of intracellular content [107]. Silver NPs coated with ceragenins, or other cationic antimicrobials, were found to be eight times more effective against bacteria than silver NP alone [108], while ceragenin-functionalized magnetic NPs were shown to impede not only bacterial biofilm formation but also of fungal ones [109,110]. Despite the great promise of these AMP mimics in terms of long-term stability, low toxicity, and antibiofilm activity towards a great number of pathogens, their use for coatings is scarcely reported. Bulk ceragenins have been incorporated into contact lenses or used as a coating on fracture fixation plates to prevent *P. aeruginosa* and *S. aureus* biofilm establishment in vitro and in vivo [111,112]. Hashemi et al. demonstrated that ceragenin-protected endotracheal tubes resisted microbial colonization, decreasing the adverse effects of intubation associated with infection and inflammation [113].

### 4.3. Marine-Derived Antibacterial Lipids

Medicinal plants and marine organisms are natural sources of many antimicrobial compounds. Plant-derived phenolics, diterpenes/terpenoids, alkaloids, sulfur-containing compounds, glycosides, and fatty acids, “generally recognized as safe” (GRAS), have shown strong antibacterial activity towards Gram-positive and Gram-negative bacteria and low probability for triggering AMR [114]. Slow-moving or sessile marine organisms produce antimicrobial molecules (e.g., fatty acids and peptides) as a part of their adaptive defense mechanisms to protect themselves against pathogens including bacteria, viruses, and fungi.

Fatty acids, monoacylglycerols, sterols, and terpene derivatives are among the most studied antimicrobial lipid classes. Their strong antimicrobial efficiency against a wide spectrum of microorganisms is related to their chemical structure and depends on the acyl chain length, the stereochemistry, the degree of unsaturation, and the esterification [115]. Long-chain polyunsaturated fatty acid combined with benzoyl peroxide synergistically inhibited the growth of *S. aureus* due to the increase in the bacterial membrane permeability that improved the penetration of the bactericidal active [116]. Lipids have been mainly used to produce vehicles for delivery of conventional antibiotics both due to their membrane destabilization potential and resemblance to natural membranes. For instance, lipid-polymer NPs loaded with antibiotics demonstrated higher antibiofilm activity on *P. aeruginosa* than the polymer NPs alone [117]. Solid-lipid NPs encapsulating rifampin were used against *S. epidermidis*, destroying the biofilm and affecting the bacterial viability [118]. Recently, the use of solely lipid-based NPs has gained attention, avoiding the use of antibiotics. For example, Rozenbaum et al. produced monolaurin lipid NPs that were able to reduce the bacterial concentration of methicillin-resistant *S. aureus* inside biofilms in vitro and the activity synergistically increased when AMPs were absorbed onto the NPs. However, these effects were not observed in wound models in mice [119].

### 4.4. Bactericidal Enzymes

Above, we discussed the ability of some enzymes to target QS and destroy the components of EPM; however, other enzymes display a direct antimicrobial effect, mainly hydrolyzing components of the cell wall or by catalyzing the formation of oxidative molecules. Lysozyme is a muramidase that catalyzes the hydrolysis of β-1,4-linkages between *N*-acetyl-d-glucosamine and *N*-acetylmuramic acid and was loaded into mesoporous NPs and magnetite-chitosan NPs [120,121]. Wang et al. produced gelatin composites for wound-healing applications, carrying loaded mesoporous polydopamine NPs with lysozyme that were able to disperse *E. coli* biofilm [122].

Cellobiose dehydrogenase (CDH) is another enzyme that elicits bactericidal action through the production of hydrogen peroxide. CDH has been shown to inhibit the growth of a panel of multidrug-resistant pathogens including *E. coli*, *S. aureus*, *S. epidermidis*, *P. mirabilis*, *S. maltophilia*, *Acinetobacter baumannii*, and *P. aeruginosa* thanks to the oxidation of bacterial extracellular polysaccharides. CDH NPs have been also produced in situ and subsequently deposited onto silicone surfaces using ultrasound, effectively reducing viable *S. aureus* cells and the total amount of deposited biomass (Table 1) [123]. In more recent work, antibacterial CDH and matrix-degrading DNAse were simultaneously immobilized onto chitosan NPs, which penetrated the biofilm structure and acted synergistically on preformed multi-species biofilms (Figure 3C) [124]. Though aside from the aforementioned general limitations of enzyme stability, the efficacy of such strategies could be affected by potential inflammatory response, associated with oxidative stress.

### 4.5. Metal and Metal Oxide Nanoparticles

Metal and metal oxide NPs are stronger antimicrobial agents, compared to ionic metals or metal macrostructures. Their small size and high area-to-volume ratio improve the penetration and interaction with the bacterial membrane, enhancing their antimicrobial properties. The unspecific mechanisms of antibacterial action such as disruption of the membrane stability, ROS production, and release of metal ions generally hinder the appearance of AMR. However, it is important to note that metal NPs have been reported to induce toxicity in mammalian cells. The most used metals for NPs synthesis are silver, gold, and zinc oxide. Metal NPs have been coated on catheters and implants to avoid biofilm formation or loaded in wound dressings for elimination of established biofilms in chronic wounds (Figure 3B) [125,126,127,128,129,130,131,132,133,134,135,136,137,138,139,140]. Selenium NPs were able to eradicate *S. aureus*, *P. aeruginosa*, and *Salmonella typhi*, disrupting their cell walls, in addition, cotton fabrics functionalized with Se NPs avoid the formation of *E. coli* and *S. aureus* biofilm on their surfaces (Table 1) [141,142]. Other metal NPs that have displayed antibiofilm properties are titanium dioxide, copper, and nickel [143,144,145,146,147]. Our group has a wide experience in the coating of medical devices with metal and metal oxide NPs, individually or in combination with other bioactive macromolecules. This is a proven strategy to reduce the intrinsic metal toxicity and/or to enhance the antimicrobial performance. Zinc oxide NPs were sonochemically coated on cotton medical textiles using laccase-oxidized gallic acid as a bioadhesive. The coating exhibited antimicrobial activity towards *S. aureus*, which was not affected even after 20 washing cycles at 75 °C [148]. Similarly, contact lenses were coated using ultrasound with zinc oxide NPs, chitosan, and gallic acid, displaying contact-killing capacities towards *S. aureus*, without affecting the optical properties [149]. Finally, silver NPs decorated with aminocellulose or chitosan were used to synthesize layer-by-layer hyaluronic acid coatings and membranes that inhibited completely bacterial growth and reduced biofilm formation (Table 1) [30].

**Table 1:** Relevant examples of nanomaterials and coatings against biofilms.

Strategy	Actives	Nanoparticle	Antibiofilm Activity	Application	Ref.
**Quorum sensing inhibitors**	Quorum sensing inhibitors	Gold-silica-cinnamaldehyde	*S. aureus*	Colloidal suspension	[38]
		Acid-poly (lactic-*co*-glycolic acid)-azithromycin	*P. aeruginosa*	Colloidal suspension	[44]
		Eugenol nanoemulsion	*E. coli*	Nanocomposite hydrogel	[48]
	Quorum-quenching enzymes	Silver-aminocellulose-acylase	*P. aeruginosa*	Colloidal suspension	[33]
		Acylase-gentamicine			[28]
		Gold-lactonase			[56]
	Metal and metal oxides	Gold, nickel oxide, tellurium and selenium	*P. aeruginosa*	Colloidal suspension	[59,60,62]
	Mimics of QS machinery	CAI-1 (autoinducer)	*V. cholera*	Colloidal suspension	[64]
		Molecularly imprinted NPs	*P. aeruginosa*		[66]
**Anti-adhesion**	NO donors	NO-releasing silica NPs	*P. aeruginosa*, *E. coli*, *S. aureus*, *S. epidermidis*	Colloidal suspension	[70]
		Polymeric stars-spermine	*P. aeruginosa*	Colloidal suspension	[71]
		Silver-NO donor	*P. aeruginosa*, *E. coli*, *S. aureus*, *K. pneumoniae*	NP-containing PVA/PEG-films	[73]
	Zwitterions	PSBMA-silver, PCBDA-silver	*P. aeruginosa*, *E. coli*, *S. aureus*	Membranes and gauzes coatings	[77,80]
	Matrix degrading enzymes	Silver-amylase	*S. aureus*, *E. coli*	Colloidal suspension	[34]
		Zinc oxide-amylase		Catheters coatings	[31]
		Shellac NPs-protease	*S. aureus*	Colloidal suspension	[88]
**Bactericidal**	Peptides	RBRBR-chitosan, IVFK-silver	*S. aureus*, *E. coli*	Colloidal suspension	[95,96]
		Polymyxin B-PSBMA	*P. aeruginosa*	Catheters coatings	[100]
	Antibacterial enzymes	Lysozyme-magnetite-chitosan	*S. aureus*, *P. aeruginosa*	Colloidal suspension	[121]
		Lysozyme-polydopamine	*E. coli*	Hydrogel dressing	[122]
		Cellobiose dehydrogenase	*S. aureus*	Surface coatings	[123]
	Metal and metal oxides	Silver	*S. aureus*, *E. coli*, *P. aeruginosa*	Catheters and implants coatings	[125,126,127]
		Silver-chitosan	*S. aureus*, *E. coli*	Coatings	[30]
		Selenium	*S. aureus*, *P. aeruginosa* and *S. typhi*	Colloidal suspension	[141]
		Zinc oxide-chitosan-gallic acid	*S. aureus*	Contact lenses coatings	[149]

## 5. Conclusions

Different nanotechnological approaches have been used for highly efficient strategies against biofilms. We have presented how antimicrobial and antibiofilm actives can be engineered for three different strategies: (i) disrupting the QS, (ii) preventing the bacterial attachment and promoting the biofilm detachment, and (iii) killing the bacteria encased inside the biofilm. Enzymes, depending on their type and activity, can quench QS, destroy selected components of the EPM, or directly exercise an antimicrobial role; however, their stability and possible side effects in vivo may limit their use. Metal NPs are excellent antimicrobial agents and have been reported to interfere in the intercellular communication; however, their intrinsic toxicity requires innovative approaches for their formulation. Zwitterions are able to prevent bacterial colonization of surfaces, while other molecules such as lipids and peptides directly disrupt the bacterial membrane, regardless of whether in planktonic or biofilm form. We showed how the combination of antibiofilm and antimicrobial agents and their synergistic activity are critical for the efficacy. However, although the numerous examples in this fruitful field display highly promising results, they are usually restricted to in vitro models in idealized laboratory conditions. Thus, the immense progress in the lab still needs to be validated in relevant environments such as in vivo experiments and realistic settings, provided the diverse nature of clinical isolates and their capacity to develop AMR in short time. At the same time, further factors such as scale of production, economic viability, and environmental impact have to be addressed before translation into actual practice.

## Data Availability

Not applicable.
